# Elevating Research Transparency in Ayurveda: Editorial Insights on the Consolidated Standards of Reporting Trials (CONSORT) 2025

**DOI:** 10.7759/cureus.92742

**Published:** 2025-09-19

**Authors:** Satyajit P Kulkarni, Pallavi S Kulkarni, Sachin S Chandaliya, Sainath B Sitawar, Naresh Nimbalkar

**Affiliations:** 1 Panchakarma, Manjushree Research Institute of Ayurvedic Science, Gujarat Ayurved University, Gandhinagar, IND; 2 Medicine, Datta Meghe Institute of Higher Education and Research, Wardha, IND; 3 Agadtantra, Manjushree Research Institute of Ayurvedic Science, Gujarat Ayurved University, Gandhinagar, IND; 4 Panchakarma, Shri Ayurved Mahavidyalaya, Nagpur, Maharashtra University of Health Sciences, Nashik, IND; 5 Rasashastra, Bhaisaheb Sawant Ayurved Mahavidyalaya, Maharashtra University of Health Sciences, Nashik, IND; 6 Kayachikitsa, Chhatrapati Shahu Maharaj Shikshan Sanstha (CSMSS) Ayurved Mahavidyalaya, Aurangabad, IND

**Keywords:** arrive, ayurveda, clinical trial reporting, consort guidelines, research transparency, spirit guidelines

## Abstract

The release of the novel Consolidated Standards of Reporting Trials (CONSORT) 2025 and Animals in Research: Reporting In Vivo Experiments (ARRIVE) guidelines signifies a transformative shift in the landscape of clinical trial reporting. For the fields of Ayurvedic and herbal medicine, these revisions present a structured framework poised to enhance transparency, reproducibility, and global credibility in research.

This editorial aims to delineate the key updates introduced within the revised CONSORT and ARRIVE frameworks and discuss their critical implications for the conduct and reporting of Ayurvedic research.

The editorial underscores the imperative of seamless protocol integration, reinforced ethical clarity, and widespread academic adoption of these updated guidelines within the Ayurvedic research community.

## Editorial

With the growing global recognition of integrative medicine, it has become imperative for traditional medical systems, such as Ayurveda, to conform to international benchmarks for research transparency. The recently updated Consolidated Standards of Reporting Trials (CONSORT) - Animal Research: Reporting In Vivo Experiments (ARRIVE) guidelines are poised to transform clinical trial reporting in complementary medicine through the introduction of expanded checklist items, improved protocol harmonization, and stricter ethical mandates. The differentiating factors between CONSORT 2010 and CONSORT 2025 are tabulated below (Table 1).

**Table 1:** Comparative table: CONSORT 2010 vs. CONSORT 2025. CONSORT: Consolidated Standards of Reporting Trials; ARRIVE: Animal Research: Reporting of In Vivo Experiments; SPIRIT: Standard Protocol Items: Recommendations for Interventional Trials.

Feature	CONSORT 2010	CONSORT 2025
Integration	Separate guidelines	Merged with ARRIVE
Adverse Events	Optional reporting	Standardized and mandatory
Intervention Details	General description	Comprehensive delineation
Blinding	Basic guidance	Detailed methodology
Analysis Reporting	Standard	Enhanced clarity
Protocol Alignment	Implicit	Aligned with SPIRIT
Checklist and Elaboration	Static	Revised with examples
Extensions	Available	Integrated and centralized
Guideline Nature	Static	Living document
Data Sharing	Optional	Mandatory disclosure
Funding and Conflicts	Expected	Mandatory

Key revisions relevant to Ayurveda include: structured intervention reporting, which now requires detailed documentation of experimental and comparator interventions, including administration methods and co-interventions, especially vital for complex Ayurvedic therapies [1]. Harmonization with Standard Protocol Items: Recommendations for Interventional Trials (SPIRIT) guidelines ensures coherent trial protocols from conception to analysis [2]. New standards address challenges in blinding traditional therapies, offering detailed guidance or justified transparency when blinding is not feasible [3]. Clear articulation of analysis sets, missing data handling, and subgroup analyses improves reliability [4]. Thus, CONSORT 2025 functions as a dynamic document with periodic updates and a shared portal with SPIRIT for resources and examples [5].

Discussion

Adopting CONSORT 2025 offers a robust pathway to elevate the rigor and credibility of Ayurvedic research [6]. First, mandatory clinical trial registration is essential to minimize reporting biases and enhance transparency. Although the Clinical Trials Registry-India accommodates traditional medicine trials, its current dataset is primarily designed for conventional medicine [7]. This underscores the need for customized registration frameworks tailored to Ayurvedic interventions, which often involve complex, multi-component therapies [1].

Second, transparent reporting significantly improves the reproducibility of findings and strengthens the foundation for systematic reviews and meta-analyses. By adhering to comprehensive standards, Ayurvedic research can transition from anecdotal documentation to evidence-based synthesis, thereby increasing its scientific validity and global relevance [8].

Third, ethical integrity must be upheld, particularly in non-pharmacological interventions such as Panchakarma, where blinding and placebo controls pose unique challenges. The revised CONSORT guidelines emphasize ethical clarity and advocate for justified transparency when conventional blinding is not feasible. Ethics committees in Ayurveda, Yoga and Naturopathy, Unani, Siddha, and Homeopathy (AYUSH) institutions play a pivotal role in ensuring patient safety and responsible research conduct [9].

Fourth, adherence to standardized reporting guidelines enhances journal acceptance and scholarly impact. Transparent disclosure of methodology and data fosters trust and improves citation metrics. Journals endorsing CONSORT often demonstrate higher reporting quality, and articles conforming to these standards are more likely to be cited and recognized within the academic community [10]. This increased visibility and credibility can attract further funding and collaborative opportunities, accelerating the integration of Ayurvedic insights into mainstream healthcare paradigms. Finally, the structured framework provided by CONSORT 2025 encourages a move toward more rigorous research methodologies within Ayurveda, fostering a culture of systematic inquiry that aligns with global scientific standards while preserving the unique ontological and epistemological foundations of the traditional system. The integration of such rigorous methodologies would facilitate a deeper understanding of Ayurvedic principles through empirically validated outcomes, bridging the gap between traditional wisdom and contemporary scientific discourse.

Empirical observations indicate that many Ayurvedic clinical trials, particularly those conducted within academic settings, frequently exhibit significant methodological limitations. These include their predominantly single-centered nature, insufficient sample sizes, lack of reporting of attrition rates or adverse drug reactions (ADRs), and a notable absence of trial registration [11,12]. Furthermore, common shortcomings involve the lack of a standardized comparator group, inadequate reporting of attrition rates, and insufficient documentation of adverse drug events. A significant concern is the apparent lack of awareness within Institutional Ethics Committees regarding these fundamental flaws, which compromises the ethical oversight and scientific rigor of such studies [13].

While there have been some improvements in the quality of Ayurvedic trials, the pace of these advancements remains notably slow and insufficient to address the systemic issues. Therefore, there is an urgent imperative to elevate the quality of academic research in Ayurveda. This necessitates not only the mandatory adoption of guidelines like CONSORT 2025 for AYUSH-funded projects, comprehensive training workshops for postgraduate and PhD guides and scholars, and strong endorsement policies from Ayurveda-focused publications, but also the establishment of a dedicated monitoring body to ensure adherence to research best practices and foster continuous quality improvement.

Finally, integrating CONSORT principles into AYUSH education is vital for cultivating a research-oriented mindset. Formal training in evidence synthesis and biomedical research methodology will empower future Ayurvedic scholars to conduct and report high-quality clinical trials, aligning traditional wisdom with modern scientific rigor.

Conclusion

To enhance research transparency in Ayurveda and elevate its global impact, the adoption of robust guidelines like CONSORT-ARRIVE 2025 is paramount. This move is crucial given the documented methodological shortcomings in many Ayurvedic clinical trials, including issues such as small sample sizes, single-center designs, and inadequate reporting of trial registration, attrition rates, and adverse drug events. To accelerate this essential shift, Institutional Ethics Committees must be better informed about these guidelines and the common flaws in Ayurvedic research. Furthermore, establishing a dedicated monitoring mechanism is vital to ensure effective implementation and continuous improvement in research quality within the field.
